# Long non-coding RNAs regulation of therapeutic resistance

**DOI:** 10.20517/cdr.2019.58

**Published:** 2019-09-19

**Authors:** Susan Tsang, Tajhal Patel, Jason T. Yustein

**Affiliations:** ^1^Integrative Molecular and Biomedical Sciences Program, Baylor College of Medicine, Houston, TX 77030, USA.; ^2^Department of Pediatrics, Texas Children’s Cancer Center, Baylor College of Medicine, Houston, TX 77030, USA.

**Keywords:** Long non-protein coding RNA, cancer, chemotherapeutic resistance, target therapeutics, hormone therapy, and microRNA sponge

## Abstract

Non-protein coding RNAs have emerged as a regulator of cell signaling and cancer progression through regulation of cell proliferation, metastatic burden, and cancer stem cell capacity. A subtype of non-protein coding RNA is long non-protein coding RNA (lncRNA). Besides their aforementioned roles in cancer cell biology, dysregulation of lncRNAs contribute to resistance to therapeutic treatments. A couple of important therapeutic classes are chemotherapy and targeted/hormone therapies. This review highlights the variety of malignancies affected by lncRNA dysregulation and the underlying mechanism causing therapeutic resistance.

## Introduction

Cancer treatment has advanced a great deal over time with the introduction of targeted therapy, improved surgical procedures, precise radiotherapy, and continual development of chemotherapy. Cancer therapy discoveries first began in the 1940’s, with the development of methotrexate^[1,2]^. Since then research has led to the production of more than a 100 chemotherapies and a variety of targeted therapeutics^[3,4]^. Some therapies like cisplatin, interferes with DNA repair while others like methotrexate acts as an inhibitor dihydrofolate reductase^[3,5]^. However, even with the availability of multiple therapies, therapeutic resistance remains a major clinical problem.

Cancer can attain drug resistance through acquired and intrinsic factors. Intrinsic factors consist of endogenous gene dyregulation, thus cancer cells are able to avoid cell toxicity when under cancer treatment. Acquired factors are activated after administration of drug treatment causing the remaining viable cells to develop a molecular perturbation which induces these cells to develop therapeutic resistance. Intrinsic and acquired factors attained within the tumor and/or in the tumor microenvironment can cause reduction in the cancer’s sensitivity to therapeutics^[6]^.

Non-protein coding RNA (ncRNA) is a subclass of RNA previously considered as non-functional RNA based upon the idea that ncRNA lacks a cellular function^[7]^. However, in recent decades research has contradicted this idea by providing evidence of ncRNA differential expression in a variety of cell types and their ability to control a multitude of cellular function(s)^[7]^. The advent of RNA sequencing, has allowed for identification and additional characterization of ncRNAs which have led to subcategorization of ncRNAs into microRNA (miRNA), lncRNA, and circular RNA (circRNA)^[8]^.

LncRNAs are 200 or more base pairs in length^[9,10]^. A proportion of lncRNAs can present genomic features seen on mRNA such as a 5’ cap and/or 3’ poly (A) tail as well as share mRNA transcriptional regulation such as lncRNAs dependence on RNA polymerase II^[10,11]^. Cellular analysis of lncRNAs has provided insight on their ability to affect cellular function such as directly impacting protein translation through recruiting ribosomal subunits, and their role as a microRNA sponge^[10]^. In relation to cancer research, lncRNAs have been shown to be both pro-oncogenic and tumor suppressive. With their ability to directly regulate mechanisms involved in response to therapeutic agents. LncRNA expression can be tissue- specific making them an attractive target for therapeutic development since tissue-specific therapies will help minimize drug induced side effects^[12]^. This review will focus on the extensive evidence supporting lncRNAs regulatory role in therapeutic resistance across multiple cancer types [Table 1].

**Table 1:** lncRNAs with the capability of perturbing chemotherapeutic resistance

lncRNA	Level of lncRNA in therapeutic resistant state	Cancer	Therapeutic agent	Mechanism of action	Ref.
ARSR	Upregulated	Liver	Doxorubicin	Binding to PTEN mRNA causing degradation of PTEN mRNA leading to enhancement of PI3K/AKT	[13]
ATB	Upregulated	Breast	Trastuzumab	Binding to miR-200c modulates ZEB1 and ZNF1 expression	[14]
BCAR4	Upregulated	Breast	Tamoxifen	Phosphorylates ERBB2 and ERBB3 leading to activation of AKT kinase 1/2	[15]
CASC2	Downregulated	Gastric	Cisplatin	Sponging of miR-19a thus decreasing apoptosis	[16]
FAM84B-AS	Upregulated	Gastric	Cisplatin	Preventing Bax translocation from cytoplasm to mitochondria and keeping cytochrome C from releasing in mitochondria thus reduction of apoptosis	[17]
FOXC2-AS1	Upregulated	Osteosarcoma	Doxorubicin	Acting on ABCB1	[18]
FOXD2-AS1	Upregulated	Bladder	Gemcitabine	Sponging of miR-143 leading to upregulation of ABCC3	[19]
GAS5	Downregulated	Breast	Trastuzumab	Interacting with miR-21 increasing PTEN	[20]
GBCDRlnc1	Upregulated	Gallbladder	Doxorubicin	Interacting with PGK1 prevents PGK1 ubiquination leads to subsequent enhancement of ATG5-ATG12	[21]
H19	Upregulated	Breast	Paclitaxel	Reducing p-AKT driving apoptotic pathway	[22]
	Upregulated	Glioblastoma	Temozolomide	Activating Wnt/β-Catenin pathway	[23]
HANR	Upregulated	Liver	Doxorubicin	Binding to GSKIP and decreasing p-GSK2Β thus reducing apoptosis	[24]
HIF1A-AS2	Upregulated	Bladder	Cisplatin	Increasing HMG1 allows for increased binding to p53, p63, and p73 which decreases apoptosis	[25]
HOTAIR	Upregulated	Breast	Tamoxifen	Binding to ER and activating of GREB1, TFF1, and c-Myc	[26]
	Upregulated	Colorectal	Cisplatin	Sponging of miR-203a-3p leads to activation of Β-Catenin/Wnt pathway	[27]
	Upregulated	Lung	Crizotinib	Inducing of ULK1 phosphorylation leading to autophagy	[28]
HOXD-AS1	Upregulated	Glioblastoma	Cisplatin	Interacting with miR-204 leading to reduction of apoptosis genes caspase-3 and caspase 9	[29]
	Upregulated	Prostate	Paclitaxel; Bicalutamide	Binding to WDR5 causes activation of PLK1, AURKA, FOXM1, CDC25c, UBE2C, CCNA2, and CCNB1	[30]
LBCS	Downregulated	Bladder	Cisplatin and Gemcitabine	Prevents binding to hnRNPK-EZH2 leading to increase in SOX2 thus reducing apoptosis	[31]
LET	Downregulated	Bladder	Gemcitabine	Increase in NF90 leading to suppressing miR-145	[32]
LINC00460	Upregulated	Lung	Gefitinib	Acting on miR-769-5p-EGFR axis	[33]
Linc00518	Upregulated	Prostate	Paclitaxel	Binding to miR-216-5p leads to enhancement of GATA6	[34]
LUCAT1	Upregulated	Osteosarcoma	Methotrexate	LUCAT1 3’ UTR region binds to miR-200c preventing competitive inhibition of miR-200c binding to ABCB1	[35]
MACC1	Upregulated	Gastric	Oxaliplatin and 5-FU	MACC1 level is dependent upon TGFB1 from mesenchymal stem cells. MACC1 binds to miR-145-5p	[36]
MALAT1	Upregulated	Colorectal	Oxaliplatin	Binding to miR-218, leading to enhances EZH2 and E-Cadherin	[37]
MBNL1-AS1	Downregulated	Lung	Gefitinib and Cisplatin	Sponging miR-301b-3p, increasing the levels of TGFBR2 to activated TGF-β	[38]
MEG3	Downregulated	Lung	Cisplatin	Inactivating of p53 and Bcl-xl, preventing mitochondrial apoptosis	[39]
NEAT1	Upregulated	Liver	Sorafenib	Suppressing miR-335 causing a decrease in c-MET	[40]
	Upregulated	Osteosarcoma	Cisplatin	Knockdown of miR-34-c causing a decrease in cell cycle arrest	[41]
	Upregulated	Prostate	Docetaxel	Binding to miR-34a leads to enhancement of RET	[42]
OIP5-AS1	Upregulated	Osteosarcoma	Cisplatin	Decrease in miR-34-5p causes elevated levels of LPAAT-Β leading to inactivation of PI3K/AKT/mTOR pathway	[43]
PVT-1	Upregulated	Colorectal	5-FU; Cisplatin	Regulating ABCB1, Bcl-2, and mTOR; increase ABCB1, MDPR1 and Bcl-2 but decreasing Bax and cleaved caspase 3	[44,45]
	Upregulated	Gastric	5-FU	Increasing Bcl-2	[46]
SBF2	Upregulated	Glioblastoma	Temozolomide	Sponging miR-151a-3p causing reduction of XRCC4	[47]
SNHG1	Upregulated	Liver	Sorafenib	miR-21 enhances SNHG1 causing nuclear retention and upregulation of SLC3A2 and enhancement of AKT pathway	[48]
SNHG12	Upregulated	Lung	Cisplatin, Paclitaxel, and Gefitinib	Binding directly to miR-181-a causing an increase in phosphorylated MAPK1 which activates MAPK1, MAP2K1, and SLUG pathway thus reducing apoptosis	[49]
TATDN1	Upregulated	Lung	Cisplatin	Sponging miR-451 leading to enhancement of TRIM66	[50]
THOR	Upregulated	Gastric	Cisplatin	Binding to 3’UTR of SOX9 leading to SOX9 mRNA stability	[51]
TP73-AS1	Upregulated	Glioblastoma	Temozolomide	Loss of ALDH1A1	[52]
TUG1	Upregulated	Colorectal	Methotrexate	Interacting with miR-186 enhances CPEB2	[53]
	Upregulated	Liver	Adriamycin	Targeting ABCB1, PARP, and caspase-3	[54]
UCA1	Upregulated	Bladder	Cisplatin	Upregulation of WNT6 pathway	[55]
	Upregulated	Breast	Tamoxifen	Activating Wnt/β-Catenin; p-AKT/mTOR	[56]
	Upregulated	Colorectal	5-FU	Sponging of miR-204-5p leading to upregulation of Bcl-2, RAB22A, and CREB1	[57]
	Upregulated	Lung	Gefitinib	Inducing AKT/mTOR pathway	[58]
	Upregulated	Prostate	Docetaxel	Reducing miR-204 which increased SIRT1	[59]
XIST	Upregulated	Colorectal	Doxorubicin	Binding to miR-124 leading to an increase in SGK1	[60]
ZFAS1	Upregulated	Gastric	cis-platinum and Paclitaxel	Enhancing Wnt/β-catenin pathway	[61]

ARSR: Activated in RCC with sunitinib resistance; PTEN: phosphatase and tension homolog; PI3K: phosphoinositide 3-kinase; AKT: protein kinase 3; ATB: activated by TGF-β; ZEB1: Zinc finger E-Box binding homeobox 1; ZNF1: Zinc finger protein 1; BCAR4: breast cancer anti-estrogen resistance 4; ERBB2: Erb-B2 receptor tyrosine kinase 2; ERBB3: Erb-B2 receptor tyrosine kinase 3; CASC2: cancer susceptibility candidate 2; Bax: Bcl-2 associated X; FOXC2-AS1: cancer susceptibility candidate 2; ABCB1: ATP bonding cassette subfamily B member 1; MDPR1: multi drug resistance protein 1; FOXD-AS1: FOXD2 adjacent opposite strand RNA 1; ABCC3: ATP binding cassette subfamily c member 3; GAS5: growth arrest-specific transcript 5; GBCDRlnc1: gallbladder cancer drug resistance-associated lncRNA1; PGK1: phosphoglycerate kinase 1; HANR: HCC associated long non-coding RNA; GSKIP: glycogen synthase Kinase 3 interacting protein; GSK3β: glycogen synthase kinase 3 β; HIF1A-AS2: hypoxia inducible factor 1 alpha-antisense RNA 2; HMG1: high mobility group Box 1; HOTAIR: HOX transcript antisense RNA; ER: estrogen receptor; GREB1: growth regulating estrogen receptor binding 1; TFF1: trefoil factor 1; ULK1: Unc-51 like autophagy activating kinase 1; HOXD-AS1: HOXD cluster antisense RNA 1; PKL1: kinesin-like protein Pkl1; AURKA: Aurora kinase A; FOXM1: Forkhead Box M; CDC25c: cell division cycle 25c; UBE2C: ubiquitin conjugating enzyme E2 C; CCNA2: cyclin A2; CCNB1: cyclin B1; LBCS: low expressed in bladder cancer stem cells; hnRNPK: heterogeneous nuclear ribonucleoprotein K; EZH2: enzyme of zeste 2 polycomb repressor nuclear complex 2 subunit; SOX2: SRY-box2; LET: low expression in tumor; EGFR: epidermal growth factor receptor; GATA6: GATA binding protein 6; LUCAT1: lung cancer associated transcript 1; MACC1: metastasis associated in colon cancer-1; 5-FU: 5-fluorouracil; MALAT1: metastasis associated lung adenocarcinoma transcript 1; MBNL1-AS1: muscleblind-like 1 antisense RNA 1; TGFBR2: transforming growth factor beta receptor 2; TGF-β: transforming growth factor β; MEG3: maternally expressed 3; Bcl-XL: B-cell lymphoma-extra large; NEAT1: nuclear enriched abundant transcript 1; c-MET: MET proto-oncogene; RET: ret proto-oncogene; OIP5-AS1: OIP5 antisense RNA 1; LPAAT-B: lysophosphatidic acid acyltransferase B; AKT: protein kinase 3; mTOR: mammalian TORC1; PVT-1: plasmacytoma variant transcript 1; Bcl-2: B-cell lymphoma 2; SBF2: SBF2 antisense RNA 1; XRCC4: X-ray repair cross complementing 4; SNHG1: small nucleolar RNA host gene 1; SLC3A2: solute carrier family 3 member 2; SNHG12: small nucleolar RNA host gene 12; MAPK1: mitogen-activated protein kinase 1; MAP2K1: mitogen-activated protein kinase kinase 1; SLUG: snail family transcriptional repressor 2; TATDN1: TatD DNase domain containing 1; TRIM66: tripartite motif containing 66; THOR: testis associated oncogenic; SOX9: SRY-Box 9; TP73-AS1: TP73 antisense RNA 1; ALDH1A1: aldehyde dehydrogenase 1 family member A1; TUG1: taurine upregulated gene 1; CPEB2: cytoplasmic polyadenylation element binding protein 2; PARP: poly ADP ribose polymerase; UCA1: urothelial cancer associated 1; WNT6: Wnt family member 6; RAB22A: RAB22A, Member RAS Oncogene Family; CREB1: CAMP responsive element binding protein 1; SPRK1: SRSF protein kinase 1; SIRT1: NAD-dependent deacetylase sirtuin-1; XIST: X-inactive specific transcript; SGK1: Serum/Glucocorticoid Regulated Kinase 1; ZFAS1: ZNFX1 Antisense RNA 1

## Chemotherapy

A mainstay treatment for a majority of malignancies is chemotherapy. Chemotherapy causes cancer cell toxicity by disrupting DNA replication, which can lead to cell death. There are several classes of chemotherapeutic agents: alkylating agents, platinum complexes, taxanes, tubulin interactive agents, topoisomerase II inhibitors, and anthracyclines^[62]^. While chemotherapy is the first-line treatment for a majority of cancers, resistance to chemotherapy is a major obstacle in cancer treatment. Common reasons for cancer resistance is the tumor cells are able to minimize their uptake of the drug and/or the enhance release of the drug from the cell at a rate where cell toxicity does not occur^[63]^.

Alkylating agents principal role is to control DNA synthesis in cells which are undergoing proliferation and in turn causing cytotoxicity. Alkylating agents can affect DNA synthesis by adding an alkyl group to guanine bases of DNA thus inhibiting double helix to properly form^[64]^. Glioblastoma is the leading cause of central nervous system brain tumor death in adults^[65]^. One of the first-line therapeutics to treat this disease is an alkylating agent, temozolomide (TMZ)^[65]^. TMZ is an alkylating agent that can cause cell cycle arrest by adding a methyl group to purine bases of DNA^[65]^. Unfortunately there are inherent and acquired factors which prevent glioblastoma cells from being sensitive to TMZ.

There have been several lncRNAs that dysregulate glioblastoma’s sensitivity to TMZ. One being lncRNA-TP73-AS1; clinical data has correlated elevated levels of TP73-AS1 to poor prognosis for glioblastoma patients. Analysis looking into glioblastoma cancer stem cells (gCSC) determined TP73-AS1 is higher in gCSC than compared to primary glioblastoma tissue. In addition knockdown of TP73-AS1 in gCSC suppresses cancer stem cell marker ALDH1A1^[52]^. An inherent characteristic of gCSC is their ability to enhance tumor-driving therapy resistance, which is the reason why further studies were done to examine if knockdown of TP73-AS1 in gCSC will enhance gCSC sensitivity to TMZ. There hypothesis held true in that knockdown of TP73-AS1 elevated gCSC sensitivity to TMZ.

LncRNA-SBF2 antisense RNA1 (SBF2-AS1) is located within the exosome of glioblastoma tumor microenvironments and is another regulator of glioblastoma sensitivity to TMZ. SBF2-AS1 is seen to be upregulated in TMZ-resistant glioblastoma cells compared to parental cells. Functional studies found that knockdown of SBF2-AS1 in glioblastoma cells leads to diminished resistance to TMZ^[47]^. SBF2-AS1 exerts its ability to cause chemotherapeutic resistance by sponging miR-151a-3p causing a decrease in XRCC4^[47]^. The reduction of XRCC4 is suppressive to tumor progression, because XRCC4 enhances double strand repair in glioblastoma^[47]^. Additionally, lncRNA-H19 is upregulated in TMZ-resistant cells. The knockdown of H19 in the TMZ-resistant cells enhances TMZ sensitivity through activating the Wnt/β-Catenin pathway^[23]^.

Platinum based drugs are complexes consisting of neutral platinum (II) with amine ligands. Examples of platinum based drugs on the market are cisplatin, carboplatin, and oxaliplatin^[66]^. Cisplatin works as a platinum based chemotherapeutic drug by crosslinking the purine bases of DNA, which affects DNA repair thus drives apoptosis^[3]^. Platinum based agents are commonly combined with other cancer therapeutics to help circumvent therapeutic resistance^[66]^.

Cisplatin is a platinum-based chemotherapeutic drug used to treat NSCLC^[67]^. The high resistance of NSCLC to cisplatin has led to ongoing research focusing on how lncRNAs may enhance sensitivity of NSCLC to cisplatin. The lncRNA-TatD DNase domain containing 1 (TATDN1) has been shown to be upregulated in NSCLC tissue compared to normal tissue. TATDN1 is at higher levels in cisplatin-resistant NSCLC cells compared to cisplatin-sensitive NSCLC cells^[50]^. Chemosensitivity assays have demonstrated that knockdown of TATDN1 leads to an increase sensitivity of NSCLC cells to cisplatin. Molecular studies have shown that regulation of chemosensitivity is dependent upon TATDN1 binding to miR-451 which leads to enhancement of TRIM66, which has oncogenic properties^[50]^. A lncRNA-small nucleolar RNA host gene 12 (SNHG12) affects NSCLC sensitivity to a variety of therapeutic drugs^[49]^. SNHG12 is overexpressed in NSCLC compared to normal lung^[49]^. In addition, NSCLC cells resistant to cisplatin had higher levels of SNHG12 than non-resistant cells. To further elucidate SNHG12 role in therapeutic sensitivity, SNHG12 was knocked down in resistant cells then treated with cisplatin. The results showed that cells with SNHG12 knockdown had lower IC_50_ values for cisplatin than the resistant cells. The regulatory axis causing SNHG12 role in drug resistance is through SNHG12 sponging miR-181-a, which causes an increase in phosphorylated MAPK1 which activates MAPK1, MAP2K1, and SLUG pathway, thus preventing apoptosis^[49]^.

The most common subtype of NSCLC is lung adenocarcinoma^[68]^. As with other types of drugs used to treat NSCLC, cisplatin resistance is a major issue in treating lung adenocarcinoma^[39]^. Upon profiling, the lncRNA-MEG3 was identified to be downregulated in cisplatin-resistant cells. Further validation found that overexpressing MEG3 can help increase lung adenocarcinoma cells sensitivity to cisplatin. It was observed that loss of MEG3 leads to inactivation of p53 and Bcl-xL, thus preventing mitochondrial apoptosis^[39]^.

While targeting lncRNAs within NSCLC would be beneficial in enhancing NSCLC sensitivity to therapeutic treatments, the targeting of lncRNAs specific to NSCLC cancer stem cells (CSC) would further increase the efficacy of these drugs. When comparing lncRNAs differentially expressed in NSCLC cells compared to NSCLC CSCs, Li *et al*.^[38]^ found lncRNA-MBNL1-AS1 had higher levels in NSCLC cells when compared to NSCLC CSCs^[38]^. Additional clinical support showed reduction of MBNL1-AS1 in NSCLC tissue of patients with lymph node metastasis *vs*. normal adjacent tissue and NSCLC tissue from patients without lymph node metastasis. Functional experiments determined that overexpression of MBNL1-AS1 can significantly reduce the IC_50_ of NSCLC CSC for cisplatin. It was recently reported that MBNL1-AS1 regulates therapeutic resistance in CSCs by competitive inhibition of miR-301b-3p. Downregulation of MBNL1-AS1 leads to enhanced levels of miR-301b-3p and higher levels of TGFBR2, which causes downstream activation of the TGF pathway thus reducing CSC phenotypes^[38]^.

The multi-modal treatment for muscle-invasive bladder cancer treatment consist of surgery, neoadjuvant chemotherapy-cisplatin, and adjuvant platinum-based chemotherapy^[69]^. The multimodal treatment for bladder cancer has lengthened the survival time but is highly ineffective at improving the overall survival rate due to therapeutic resistance^[70]^. To optimize treatment protocols, we must elucidate signaling pathways leading to resistance to therapeutics. LncRNA-UCA1 has been established in multiple cancers as oncogenic, including in bladder cancer^[71,72]^. Previous studies have identified UCA1 enhances epithelial to mesenchymal transition and invasion^[71]^. Additional functional results identified UCA1 ability to influence cisplatin resistance through upregulation of the Wnt pathway^[55]^.

As with other cancer types, bladder cancer is susceptible to molecular changes based upon oxygen intake and onset of hypoxia. It was found that hypoxic cells have an increase of hypoxia inducible factor 1 (HIF1), leading to elevated levels of lncRNA-hypoxia inducible factor 1α-antisense (HIF1A-AS2), which enhances cisplatin resistance^[25]^. HIF1A-AS2 induces this phenotype by increasing HMGA1 which leads to enhanced binding to p53, p63, and p73 causing a decrease in BAX and an overall inhibition in apoptosis. This suggest that the HIF1A-AS2/HMGA1/p53 pathway may play an important role for therapeutic targeting in hypoxia-mediated therapeutic resistance in bladder cancer^[25]^.

While the before mentioned lncRNAs were oncogenic, there are lncRNAs which possess tumor suppressor functions. LncRNA-low expressed in Bladder cancer stem cells (LBCS) was identified through stem cell profiling^[31]^. Survival analysis of bladder cancer patient data demonstrates that patients with lower quantities of LBCS exhibit poorer overall survival. In addition, LBCS is present at decreased levels in bladder cancer stem cells and cancer tissue^[31]^. Clinical data also shows a correlation of lower levels of LBCS to higher grades of bladder cancer^[31]^. Because chemotherapy resistance is a characteristic of cancer stem cells, it was important to understand LBCS sensitivity to cisplatin. Both *in vivo* and *in vitro* studies determined that overexpression of LBCS led to a decrease in bladder cancer resistance to cisplatin by elevating caspase 3/7 and an increase in apoptosis. The increase in apoptosis is due to LBCS direct binding to hnRNPK and EZH2. This complex then reduces the level of SOX2 thus enhances H2K27me3^[31]^.

LncRNA-PVT-1 has also been assessed as a regulator of chemoresistance in colorectal cancer by impacting the sensitivity of colorectal cancer cells to cisplatin^[45]^. In cisplatin resistant cells, there is a higher level of PVT-1 compared to the control cells. Knockdown of PVT-1 in the resistant cells increases the cells sensitivity to cisplatin^[45]^. Ping *et al*.^[45]^ noted that knockdown of PVT-1 can induce a decrease in multi drug resistance protein 1, ABCB1, and Bcl-2, while enhancement of apoptotic proteins Bax and cleaved caspase 3^[45]^. Another lncRNA seen to impact colorectal cancer cells sensitivity to cisplatin is lncRNA-HOTAIR. HOTAIR is shown to be upregulated in colorectal cancer tissue *vs*. normal adjacent tissue. In addition, the knockdown of HOTAIR helps increase colorectal cancer cells sensitivity to cisplatin. It is reported that HOTAIR regulates this phenotype by interaction with miR-203a-3p leading to the activation of β-Catenin/Wnt pathway^[27]^.

Even with the use of surgery and chemotherapy, the overall survival of patients with advanced gastric cancer is approximately one year^[46,73]^. In advanced stages of gastric cancer, the chemotherapeutics commonly used are cisplatin, 5-FU, and oxaliplatin^[74]^. Because of the lack of effective therapeutics there is an urgent need for new treatment options. LncRNA-zinc finger antisense 1 (ZFAS1) has been shown to regulate gastric cancer cells sensitivity to cis-platinum^[61]^. ZFAS1 levels are higher in gastric cancer compared to para-carcinoma tissue. In addition, suppression of ZFAS1 in gastric cancer cells increases gastric cancer sensitivity to cis-platinum. ZFAS1 is able to regulate gastric cancer sensitivity by decreasing NKD2 and activating the Wnt/Β-Catenin pathway^[61]^. LncRNA-FAM84B-AS has also been found to enhance cisplatin resistance in gastric cancer. FAM84B-AS is located on the antisense strand of FAM84B. While FAM84B acts a tumor suppressor, FAM84B-AS functions as an oncogene in gastric cancer^[17,75]^. FAM84B-AS is expressed at higher levels in gastric cancer cell lines and with progression of disease there is increase of FAM84B-AS level. To test FAM84B-AS role in gastric cancer sensitivity to cisplatin, a proliferation assay was performed and determined that the shRNA-FAM84B-AS gastric lines had higher rates of apoptosis than the parental cells. In addition, FAM84B-AS is able to reduce apoptosis by preventing Bax translocation from the cytoplasm to the mitochondrial membrane in turn keeping cytochrome c from releasing into the mitochondria^[17]^.

A tumor suppressive lncRNA, lncRNA-CASC2 was shown to be at lower levels in gastric cancer compared to normal adjacent tissue with additional finding that cisplatin-resistant gastric cells, BGC823 and SGC7901, have decreased levels of CASC2 compared to non-resistant cells. Kaplan-Meier survival analysis also showed a significant correlation between patients with low levels of CASC2 with poor prognosis^[16]^. To further confirm CASC2 role in chemosensitivity, cisplatin-resistant cells were transfected with CASC2 and subsequently decrease the IC_50_ of the cisplatin-resistant cells. The mechanism by which CASC2 is able to enhance resistance is through sponging of miR-19a thus decreasing apoptosis^[16]^. LncRNA-THOR is another regulator of cisplatin sensitivity for gastric cancer cells. THOR levels are higher in gastric cancer tissue in comparison to normal adjacent tissue. Functional analysis determined that knockdown of THOR in gastric lines suppresses both cancer stemness markers and phenotypes, in addition to decreasing gastric cancer cells sensitivity to cisplatin. SOX9 was a marker seen to have the largest decrease in expression when gastric cancer cells have knockdown of THOR. Mechanistically, THOR binding to the 3’ UTR region of SOX9 leads to SOX9 mRNA stability causing increase in stemness phenotypes and cisplatin resistance^[51]^.

In osteosarcoma, lncRNA-NEAT1 is a regulator of cisplatin sensitivity. With data showing NEAT1 is overexpressed in osteosarcoma tissue in comparison to non-malignant tissues. NEAT1 causes its tumorigenic effect by enhancing osteosarcoma cell viability when treated with cisplatin. Results of both *in vivo* and *in vitro* studies, found that when both NEAT1 knockdown and parental cells are treated with cisplatin, the NEAT1 knockdown line had enhanced apoptosis. It was reported that NEAT1 promotes chemotherapeutic resistance in osteosarcoma by reducing the level of miR-34c and causing a decrease in cell cycle arrest via enhanced Bcl-2 and cyclin D1 expression^[41]^. LncRNA-OIP5-AS1 is also an oncogene that enhances chemotherapeutic resistance in osteosarcoma. OIP5-AS1 was overexpressed in cisplatin-resistant osteosarcoma cells and the knockdown of OIP5-AS1 in these resistant cells led to an increase in osteosarcoma cell sensitivity to cisplatin. While it was shown that knockdown of OIP5-AS1 downregulated drug resistance genes, OIP5-AS1 reduction also enhanced the expression of miR-34-5p. Increased levels of miR-34-5p caused suppression of LPAAT-β which leads to diminished PI3K/AKT/mTOR pathway activity^[43]^.

While in glioblastoma, lncRNA-HOXD-AS1 is a factor that impacts cisplatin induced cell toxicity^[29]^. HOXD-AS1 is upregulated in glioblastoma tissue. Kaplan-Meier survival analysis demonstrates that glioma patients with high HOXD-AS1 correlates with poor survival. One of the ways HOXD-AS1 is able to promote a low overall survival is by reducing glioblastoma sensitivity to cisplatin. Cisplatin-resistant glioblastoma cancer cells have a significantly higher HOXD-AS1 level compared to parental cells. Molecular analysis identified HOXD-AS1 binding of miR-204 suppresses apoptotic genes: caspase-3 and caspase-9 leading to the lack in glioblastoma sensitivity to cisplatin^[29]^.

Oxaliplatin, is another platinum based drug^[76]^. The lncRNA-MALAT1 has been shown to be elevated in colorectal cancer tissue compared to normal tissue. In addition, advanced colorectal tumor stage positively correlates with MALAT1 expression. Furthermore, chemosensitivity studies demonstrated that the level of MALAT1 in colorectal cancer cells correlates to their resistance to oxaliplatin. MALAT1 is able to propagate this phenotype by sponging miR-218 leading to enhancement of EZH2 and E-Cadherin^[37]^.

The presence of mesenchymal stem cells (MSC) in the heterogeneous cellular structure of a tumor is a factor that enhances multi-drug resistance in gastric cancer^[77,78]^. The work of He *et al*.^[36]^ determined how MSC enhancement of a lncRNA can regulate gastric cancer sensitivity to chemotherapeutic agents. Their study found gastric cancer cells incubated with MSCs led to a reduction in gastric cancer cells sensitivity to oxaliplatin and enhanced stemness phenotypes^[36]^. Based on previous research correlating fatty acid oxidation (FAO) to stemness and chemosensitivity, the investigation focused on gastric cancer cells requires FAO induction to allow for an increase in chemoresistance^[79]^. They identified that gastric cancer cells in the presence of MSCs led to an enhancement of FAO-associated enzymes and an increase in lncRNA-metastasis-associated in colon cancer-1 (MACC1) to promote chemoresistance and stemness. The upregulation of MACC1 in gastric cancer cells was dependent upon secretion of TGFΒ1 from MSC. MACC1 subsequently binds to miR-145-5p which then enhances carnitine palmitoyltransferase 1 to induce chemoresistance and stemness behavior in gastric cancer cells^[36]^.

Taxane class of anticancer agents alters the metaphase to anaphase transition by disrupting spindle microtubule formation, thus leading to cell death. Previous literature has cited that taxane resistance can occur in tumors which contain α and Β-tubulin which polymerize into mitrotubules preventing the cell death induced by taxanes^[80]^.

Triple Negative Breast Cancer (TNBC)/basal-like sub-type makes up 15%-20% of all breast cancer diagnose^[81]^. Unlike ER+ and HER2+ breast cancer, TNBC’s are primarily treated with chemotherapy^[81]^. The first-line taxane based chemotherapeutic agent used to treat TNBC patients is paclitaxel. Paclitaxel functions by binding β-tubulin which causes microtubule stabilization and apoptotic cell death^[82]^. The lncRNA-H19 has been shown to be elevated in TNBC paclitaxel-resistant lines compared to parental cells. H19 is a lncRNA highly expressed during embryonic development but decreases after birth, specifically in mammary tissue^[83]^. Knockdown of H19 in paclitaxel resistant TNBC cell lines increases sensitivity to paclitaxel by reducing p-AKT (Ser473) driving the apoptotic pathway by decreasing Bcl-2 and enhancing cleaved caspase-3 and Bax expression^[22]^.

LncRNA-SNHG12 not only regulates NSCLC resistance to cisplatin but also to paclitaxel. NSCLC cells resistant to paclitaxel have higher levels of SNHG12 than non-resistant cells and knockdown of SNHG12 in paclitaxel-resistant lines causes a suppression of paclitaxel resistance. SNHG12 decreases sensitivity through the binding of miR-181-a leading to phosphorylation of MAPK1 thus a reduction in apoptosis^[49]^.

In gastric cancer, lncRNA-ZFAS1 regulates gastric cancer sensitivity to paclitaxel^[61]^. Gastric carcinoma cells have elevated levels of ZFAS1 compared to gastric paracarcinoma cells. The knockdown of ZFAS1 causes increase in gastric cancer sensitivity to paclitaxel by suppressing the NKD2 leading to induction of Wnt negative regulator, thus leading to the induction of Wnt/Β-Catenin pathway^[61]^.

Castration resistant prostate cancer is treated with several taxane-based therapeutics^[84]^. He *et al*.^[34]^ identified Linc00518 as elevated in prostate cancer compared to non-malignant models. In addition, paclitaxel-resistant DU145 and PC3 cells have increased levels of Linc00518 compared to non-resistant cells. With the use of siLinc00518, they determined that knockdown of Linc00518 causes increase sensitivity of prostate cancer cells DU145 and PC3 to paclitaxel. With the help of bioinformatic tools, it was predicted and then confirmed that Linc00518 binds to miR-216b-5p. Additional studies identified Linc00518-miR-216b-5p binding leads to enhancement of GATA6^[34]^. Another lncRNA which plays a role in sensitivity to paclitaxel is lncRNA-HOXD-AS1. It was seen that prostate cancer tissues from patients with higher grade prostate cancer have elevated levels of HOXD-AS1 compared to tissue from lower tumor grades. By knocking down HOXD-AS1, there is an increase prostate cancer cell sensitivity to paclitaxel. The mechanism which by HOXD-AS1 is able to regulate the sensitivity of prostate cancer cells to paclitaxel is by HOXD-AS1 binding to WDR5 leading to activation of proteins PLK1, AURKA, FOXM1, CDC25c, UBE2C, CCNA2, and CCNB1 which are critical proteins in the mitosis, cell cycle regulation, microtubule stabilization, and androgen receptor signaling pathways^[30]^.

LncRNA-NEAT1 is a critical component of the nuclear paraspeckle structure and is elevated levels in prostate cancer tissue and has the ability to enhance tumorigenic behaviors which includes decreasing tumor sensitivity to taxane based chemotherapeutic agent docetaxel^[85,86]^. NEAT1 was significantly elevated in docetaxel-resistant prostate cancer cell lines compared to docetaxel-sensitive lines. The knockdown of NEAT1 in the resistant lines caused these cells to have a decrease in their IC_50_ value for docetaxel. In addition, it was found that NEAT1 causes decrease in sensitivity by binding to miR-34a, thus resulting in the increase in RET which encodes an oncogenic receptor tyrosine kinase^[42]^.

Doxorubicin is a chemotherapeutic agent used to treat both adult and pediatric cancer. Cell toxicity caused by doxorubicin is through intercalation of DNA and inhibition of topoisomerase II-mediated DNA repair. Doxorubicin resistance has been reported through the activation of resistance-mediated genes such as ABCB1, ABCC1, and TOP2A^[87]^.

Gallbladder cancer is the fifth most-prevalent digestive malignancy and one reason for poor survival is late detection of malignancy^[88]^. Unfortunately, chemotherapy treatment is highly ineffective for patients with gallbladder cancer, which strongly contributes to the 5-year survival for gallbladder cancer being less than 10%. The lack in effective treatment causes a critical need for molecular research to further understand the genetic factors preventing chemotherapeutic efficacy^[89]^. Cai *et al*.^[21]^ investigated this issue by studying the role of lncRNA-GBCDRlnc1. GBCDRlnc1 was identified through profiling the differential level of lncRNAs in doxorubicin-resistant gallbladder cell lines *vs*. doxorubicin-sensitive lines. Verification of their profiling found that GBCDRlnc1 is expressed at higher levels in gallbladder cancer tissue than adjacent normal tissue. Their research determined that knockdown of GBCDRlnc1 in gallbladder cells causes an increase in sensitivity to doxorubicin. GBCDRlnc1 promotes this tumorigenic function by interacting with phosphoglycerate kinase 1 (PGK1) and preventing its ubiquitin-mediated degradation and subsequent enhancement of autophagy regulator ATG5-ATG12 conjugate, an enhancer of chemoresistance^[21,90]^.

Doxorubicin is also used in the treatment of colorectal cancer. Zhu *et al*.^[60]^ found that doxorubicin-resistant colorectal models have an elevated level of lncRNA-XIST compared to the parental cells. Further evaluation determined knockdown of XIST leads to lessening of doxorubicin resistance in colorectal cells. XIST is able to enhance this behavior by binding to miR-124 leading to the enhancement of Serum-and Glucocorticoid-induced Kinase (SGK1)^[60]^. SGK1 is a serine/threonine protein kinase, specifically apart of protein kinase A, G, and C family^[91]^. Previous reports have shown SGK1 is elevated in colorectal tumor and enhances tumorigenic behaviors^[60]^.

Adriamycin (doxorubicin) is a chemotherapeutic agent used to treat hepatocellular carcinoma. While chemotherapy is the first-line treatment for hepatocellular carcinoma, there is the possibility of chemotherapeutic resistance^[92]^. Yang *et al*.^[54]^ profiled the differential expression of lncRNAs in adriamycin-resistant hepatocellular carcinoma to non-resistant hepatocellular carcinoma cells. From their analysis, they determined lncRNA-TUG1 was upregulated in the resistant cells unlike the non-resistant cells. Subsequently, they found siRNA knockdown of TUG1 in adriamyacin-resistant cells restored sensitivity to this chemotherapy. Their mechanistic studies found that TUG1 is able to enhance resistance through targeting the apoptotic pathway, specifically ABCB1, PARP, and caspase 3^[54]^. In addition, clinical data found earlier stages of hepatocellular carcinoma have lower levels of lncRNA-HANR compared to later stages, and suppressing HANR expression can enhance hepatocellular carcinoma sensitivity to doxorubicin. Mechanistically HANR binds to GSKIP, which reduces p-GSK2Β thus reducing apoptosis^[24]^. LncRNA-ARSR also plays are role in hepatocellular carcinoma sensitivity to doxorubicin. ARSR is overexpressed in doxorubicin-resistant hepatocellular carcinoma cells. Both *in vivo* and *in vitro* studies using doxorubicin-resistant cells with knockdown of ARSR showed an increase in hepatocellular carcinoma cells sensitivity to doxorubicin. ARSR is able to regulate doxorubicin sensitivity by binding and leading to degradation of *PTEN* mRNA and subsequent activation of PI3K/AKT pathway^[13]^.

One lncRNA recently found to be a regulator of doxorubicin sensitivity in osteosarcoma is lncRNA- FOXC2-AS1. FOXC2-AS1 is an antisense lncRNA found to be transcribed from the negative strand of the forkhead box protein C2^[93]^. FOXC2-AS1 is upregulated in osteosarcoma cell lines and tissue. In addition, doxorubicin-resistant osteosarcoma cells have higher levels of FOXC2-AS1 compared to doxorubicin-sensitive cells. In both *in vivo* and *in vitro* studies, it was seen that siFOXC2-AS1 enhances osteosarcoma cell line sensitivity to doxorubicin. FOXC2-AS1 facilitates doxorubicin sensitivity by increasing, the drug resistance gene, ABCB1^[18]^.

Another chemotherapeutic drug is gemcitabine^[94]^. Gemcitabine inhibits DNA synthesis by addition of the nucleotide analog during the elongation phase, which halts proofreading and causes cell death^[94]^. It has been shown that patients with a higher level of lncRNA-FOXD2-AS1 has gemcitabine resistant bladder cancer. In addition, confirmatory data showed that upregulating FOXD2-AS1 enhances cancer cells resistance to gemcitabine and thus allowing for continual tumor growth^[19]^. The regulatory pathway of FOXD2-AS1 is through interaction of miR-143, thus preventing the competitive binding of miR-143 and ABCC3^[19]^. The upstream interaction between miR-143 and FOXD2-AS1 causes an enhancement of ABCC3 in bladder cancer correlates with previous reports of ABCC3 being elevated in bladder cancer tissue. In addition, these reports showed overexpression of ABCC3 causes increase in proliferation and drug resistance^[95]^.

Another lncRNA examined in bladder cancer stem cells is lncRNA-LET. LET is a downstream target of TGFΒ1 and has been shown to be downregulated in bladder cancer cells^[32]^. Those cells with low expression of LET exhibit chemoresistence to gemcitabine through the regulation of NF90/miR-145 axis^[32]^. The perturbation of this axis through LET enhancement could potentially reverse the chemotherapeutic resistance effect caused by TGFβ1.

5-Flurouracil (5-FU) is the third most common chemotherapeutic agent used for the treatment of cancer^[96]^. 5-FU works by being catabolized by dihydropyrimidine dehydrogenase leading to the end product of fluorodeoxyuridine monophosphate (FdUMP). FdUMP binds to thymidylate synthase and 5,10 methylene tetrahydrofolate causing a toxic effect by inhibiting DNA synthesis and transcription^[97]^.

Gastric cancer responds to 5-FU in about 30% of cases^[98]^. Du’s group found patients with late-stage gastric cancer have elevated levels of lncRNA-PVT-1, which coincides with previous studies showing PVT-1’s ability to regulate 5-FU sensitivity^[46]^. Chemosensitivity studies were performed to see if elevated levels of PVT-1 can increase gastric cells resistance to 5-FU. The data from this study showed overexpression of PVT-1 can suppress gastric cancer cells sensitivity to 5-FU by increasing Bcl-2 leading to an anti-apoptotic effect^[46]^. LncRNA-MACC1 previously mentioned as a regulator of gastric cancer sensitivity to cisplatin also regulates 5-FU sensitivity through upregulation of MACC1 by MSC^[36]^.

In colorectal cancer, the elevated level of PVT-1 causes inhibition of colorectal cancer cells sensitivity to 5-FU. Fan *et al*.^[44]^ determined PVT-1 regulates of ABCB1 and Bcl-2 level in addition to overexpression of PVT-1 in resistant cells leads to upregulation of mTOR^[44]^.

Sorafenib is chemotherapeutic agent which inhibits cancer by inhibiting several tyrosine kinases including VEGFR1, c-Kit, and different isoforms of Raf kinase^[99]^. While clinical data showed promising anti-tumor response in patients treated with sorafenib, the survival rate of carcinoma patients has not drastically increased since approval of sorafenib^[4]^. A reason for the lack in efficacy is due to carcinoma resistance to sorafenib^[4]^. LncRNAs have been identified as regulators of sorafenib sensitivity. Recent work by the Guo lab identified lncRNA-NEAT1 upregulation in hepatocellular carcinoma tissues compared to normal adjacent tissue^[100]^. Further analysis of NEAT1 demonstrated that NEAT1 knockdown increases hepatocellular carcinoma cells sensitivity to sorafenib. Mechanistically, this is the result of NEAT1 sponging miR-335 suppressing c-MET and AKT pathway thereby reducing apoptosis^[40]^. LncRNA-small nucleolar RNA host gene 1 (SNHG1) is upregulated in sorafenib-resistant hepatocellular carcinoma cells compared to parental lines. In hepatocellular carcinoma, sorafenib resistance is caused by miR-21 enhancement of SNHG1 which leads to SNHG1 retention in the nucleus and enhances transcription of SLC3A2 and activation of the AKT pathway^[48]^.

## Targeted therapy

Targeted therapy aims to deliver drugs specific to intracellular molecular perturbations or alteration within the tumor microenvironment. The purpose is to reduce the off-target effects which develop when treating cancer patients with other types of therapeutics. As with other cancer therapies, therapeutic resistance is an obstacle when treating patients with targeted agents^[101]^.

Non-small cell lung cancer (NSCLC) accounts for approximately 85% of lung cancer cases, with a 5-year survival of about 15%^[102]^. An agent used to treat NSCLC is gefitinib, which inhibits the tyrosine kinase activity of the epidermal growth factor receptor^[103]^. Initially gefitinib provides a measureable response for many NSCLC patients however within one year approximately 50% of patients develop resistance^[33]^. Reports show perturbation of lncRNAs diminish NSCLC sensitivity to gefitinib^[33,49]^. Ma *et al*.^[33]^ reports that gefitinib-resistant NSCLC cells have elevated levels of lncRNA-LINC00460 compared to non-resistant cells. To confirm their prediction of LINC00460 role in gefitinib resistance, they transiently knocked down LINC00460 in gefitinib-resistant cells and saw an increase sensitivity to gefitinib. This group also identified that LINC00460 regulates this action by 3’UTR of LINC00460 sponging miR-769-5p, thus enhancing EGFR protein expression^[33]^.

Besides its ability to contribute to resistance to cisplatin and paclitaxel, lncRNA-SNHG12 is also able to contribute to NSCLC resistance to gefitinib. The knockdown of SNHG12 enhances NSCLC cells sensitivity to gefitinib through the previously mentioned miR-181/MAPK1 axis^[49]^. Gefitinib resistance is also present in NSCLC CSCs. NSCLC CSCs have lower levels of lncRNA- MBNL1-AS1 compared to NSCLC cells which diminishes binding to mir-301-3b and subsequent enhancement of the TGF pathway. When MBNL1-AS1 levels are increased in NSCLC CSCs, there was a noted reduction in gefitinib resistance^[38]^.

Human epidermal growth factor receptor 2 (HER2+) breast cancer is the second most lethal subtype of breast cancer^[14]^. Trastuzumab, the first-line targeted therapeutic for HER2+ patients, targets the extra-cellular domain of the HER2 receptor to inhibit its function^[104,105]^. Although trastuzumab treatment has helped in increasing the survival of HER2+ patients, it only has an approximately 35% success rate^[106]^. Studies have shown that resistance to trastuzumab can result from HER2 gene amplification and activation of PI3K. These molecular perturbations are not detected by trastuzumab thus leading to resistance^[107]^. Trastuzumab’s low rate of success suggest a critical need to identify underlying mechanisms that enhance trastuzumab effectiveness. The Shi lab determined that HER2+ SKBR-3 parental cell line had differential lncRNA-ATB expression compared to SKBR-3 trastuzumab-resistant cells (SKBR-3/TR). ATB was upregulated in the SKBR-3/TR compared to the parental line. Furthermore, knockdown of ATB in SKBR-3/TR line increased the cells sensitivity to trastuzumab. ATB binds to mir-200c thereby modulating ZEB1 and ZNF1 expression and contributing to enhanced trastuzumab resistance^[14]^. Unlike ATB, another lncRNA, lncRNA-GAS5 has been shown to be downregulated in trastuzumab-resistant cell lines and low levels of GAS5 is associated with poor prognosis. It was reported that a dual inhibitor for both ErbB1 and ErbB2 increased expression of GAS5 causing a reversal of GAS5 ability to regulate trastuzumab sensitivity in HER2+ lines. Mechanistically, GAS5 is able to regulate HER2+ cells sensitivity by binding to miR-21and increasing PTEN expression^[20]^.

A therapeutic drug used to treat late-stage NSCLC is crizotinib^[108]^. Crizotinib is an inhibitor of receptor tyrosine kinase anaplastic lymphoma kinase (ALK) and c-Met. Cells that are positive for ALK and c-Met should be highly susceptible to crizotinib leading to apoptosis^[108,109]^. Unfortunately a subset of NSCLC patients become highly resistant to crizotinib after several rounds of treatment^[108]^. Yang *et al*.^[28]^ identified a lncRNA-HOTAIR is overexpressed in NSCLC patients and that knockdown of HOTAIR can decrease NSCLC cells resistance to crizotinib^[28]^. HOTAIR is able to accomplish this phenotype by activation of ULK1 phosphorylation leading to autophagy^[28]^.

Methotrexate is an inhibitor of dihydrofolate reductase enzyme, an enzyme critical for DNA synthesis and cell growth^[5]^. Methotrexate is a targeted therapeutic agent which cancer patients can develop resistance too. The lncRNA-TUG1 is expressed at higher levels in methotrexate-resistant colorectal cancer cells when compared to non-resistant cells. Additional evidence shows that transient knockdown of TUG1 causes increased sensitivity to methotrexate in colorectal cancer cells. TUG-1 promotes chemoresistance by sponging to miR-186, which causes enhancement of, oncogene, CPEB2^[53]^.

LncRNA-LUCAT1 is seen to be upregulated in methotrexate-resistant osteosarcoma cells compared to methotrexate-sensitive cells. The transient knockdown of LUCAT1 caused an increase in methotrexate sensitivity and also a decrease in the protein level of drug resistance related genes (ABCB1, MRP5, and MVP). Mechanistically, LUCAT1 is able to enhance chemoresistance through LUCAT1 3’ UTR region sponging miR-200c and increasing the expression of ABCB1, which is a known miR-200c target^[35]^.

## Hormone therapy

Hormone therapy is commonly used to treat both breast and prostate cancer. For breast and prostate cancer, the malignancy is dependent upon the level of steroid hormone receptors. Factors contributing to hormone therapy resistance are pre-receptor level, perturbation of hormone and receptor level, and/or post-receptor level^[110]^.

Approximately 70% of breast cancer patients have luminal A/estrogen receptor-positive (ER+) breast cancer which consists of low proliferation rate genes such as Ki67 and low levels of HER2^[111,112]^. Tamoxifen is a common hormone therapy used to treat ER+ patients. Tamoxifen is a non-steroidal anti-estrogen that competitively binds to estrogen receptor which then causes tamoxifen resistance. While tamoxifen treatment has reduced relapse by 47%, there is still a high majority of patients who inevitable have reoccurrence of their breast cancer^[113]^. Recent reports have shown breast cancer cells with elevated levels of certain lncRNAs are less responsive to tamoxifen. Tamoxifen acts by binding directly to the estrogen receptor (ER), thereby preventing the proliferative effect caused by estrogen binding to the receptor^[114]^. Godinho *et al*.^[115]^ found ER+ breast cancer cells have elevated levels of lncRNA-BCAR4^[115]^. In addition, BCAR4 induction in estrogen dependent breast cancer cell line, ZR-75-1, led to an increase resistance to tamoxifen therapy^[115]^. It has been found that BCAR4 contributes to tamoxifen resistance through phosphorylation of ERBB2 and ERBB3 resulting in activation of AKT and kinase 1/2^[15]^. Another lncRNA-HOTAIR, has been reported to be enhanced in breast cancer tumors and also decreases breast cancer cells sensitivity to tamoxifen. HOTAIR mediates tamoxifen resistance by binding directly to ER leading to the activation of downstream genes GREB1, TFF1, and c-Myc^[26]^. LncRNA-UCA1 was also recently discovered to cause tamoxifen resistance of breast cancer lines MCF7 and T47D through activation of the Wnt/β-Catenin pathway^[116]^. While others have demonstrated that tamoxifen resistant breast cancer lines LCC2 and LCC9 has elevated levels of UCA1, the tamoxifen resistance is caused by induction of p-AKT/m-TOR pathway^[56]^.

In non-castration resistant prostate cancer, patients receive androgen therapy, bicalutamide. Bicalutamide are anti-androgen agents that prevents androgen-androgen receptor binding. A common cause of resistance to bicalutamide is development of mutations within the androgen receptor thus preventing bicalutamide sensitivity^[110]^. As cited before prostate cancer tissue of higher grades have elevated levels of lncRNA-HOXD-AS1 compared to lower tumor grades. The knockdown of HOXD-AS1 caused prostate cancer cells to become more sensitive to bicalutamide. HOXD-AS1 is able to impact bicalutamide sensitivity by binding to WDR5 and regulating several important signaling pathways^[30]^.

## Conclusion

Through the use of transcriptomic analysis such as RNA sequencing, it was found that approximately 75% of the human genome is transcribed^[116]^. 2% of the RNA are then translated into protein, resulting in a majority of RNAs lacking protein capacity^[117]^. A majority of ncRNAs are lncRNAs, with a proportion of lncRNAs having a role in regulating cancer progression^[9]^. LncRNAs perturb cancer development through different facets. lncRNAs HOTAIR and MALAT1 enhance metastatic phenotypes in breast and lung cancer, while lncRNA MEG3 and MKLN1-AS1 are downregulated in colorectal cancer and both function as regulators of proliferation^[118-121]^. LncRNAs can also contribute to relapse or refractory disease through their regulation on mechanisms of therapeutic resistance.

**Figure 1:**
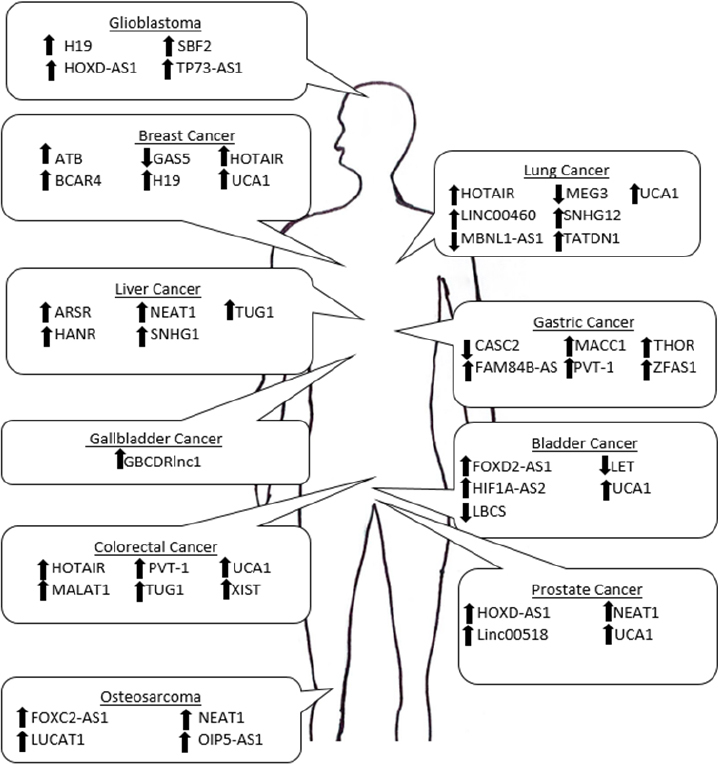
Schematic of the lncRNAs that contribute to therapeutic resistance in a variety of malignancies. The arrows represents the direction of dysregulation when cancer is in a therapeutic resistant state

For many malignancies, small molecule therapeutic treatments are the predominant course of therapy^[122]^. The development of therapeutic resistance in multiple malignancies leads to a critical need for new therapeutic strategies. The review provides an overview of dysregulated expression of critical lncRNAs and their mechanism of action driving therapeutic resistance and bringing insight to the importance of targeting this type of ncRNA to potentially increase therapeutic efficacy [Figure 1]. LncRNAs such as NEAT1, UCA1, and PVT-1 have been shown to regulate drug resistance in multiple cancer types through different molecular mechanisms. Through understanding how lncRNAs contribute to therapeutic resistance across different malignancies and therapeutic agents, it emphasizes the necessity of additional research into lncRNAs as therapeutic targets and in turn circumvent therapeutic resistance in a more expansive manner.

## Declarations

### Copyright

© The Author(s) 2019.
